# Supplementary material for: Characterization of Staphylococcus epidermidis clinical isolates from hospitalized patients with bloodstream infection obtained in two time periods
Source: PeerJ. 2022 Oct 4;10:e14030. doi: 10.7717/peerj.14030 (PMC9541613; doi:10.7717/peerj.14030)
Supplement: Supplemental Information 2 — BP: benzylpenicillin, EM: erythromycin, CM: clindamycin, Q/D: quinupristin/dalfopristin, LE: levofloxacin, GM: gentamicin, TS: trimethoprim/sulfamethoxazole, RI: rifampicin, OX: oxacillin, LI: linezolid, VM: vancomycin, CX: cefoxitin, MIC: minimum inhibitory concentration, Interp.: interpretation, R: resistant, S: sensitive. [file peerj-10-14030-s002.docx]

**Supplemental Table 1.** Antibiotic MIC values for each *S. epidermidis* clinical isolate used in this study

|  | **Antibiotics tested** | | | | | | | | | | | |
| --- | --- | --- | --- | --- | --- | --- | --- | --- | --- | --- | --- | --- |
| **Clinical Isolate** | **BP**  **(MIC)** | **EM (MIC)** | **CM**  **(MIC)** | **Q/D**  **(MIC)** | **LE**  **(MIC)** | **GM**  **(MIC)** | **TS**  **(MIC)** | **RI**  **(MIC)** | **OX (MIC)** | **LI**  **(MIC)** | **VM**  **(MIC)** | **CX (Interp.)** |
| 585 | R (≥0.5) | S (≤0.25) | S (≤0.25) | S (≤0.25) | S (≤0.12) | R (≥16) | S (20) | S (≤0.5) | R (≥4) | S (1) | S (2) | R (POS) |
| 1032 | R (≥0.5) | R (≥8) | S (≤0.25) | S (≤0.25) | S (≤0.12) | R (≥16) | R (160) | S (≤0.5) | R (≥4) | S (1) | S (1) | R (POS) |
| 1042 | R (≥0.5) | R (≥8) | R (≥8) | S (≤0.25) | S (≤0.12) | S (≤0.5) | S (≤10) | S (≤0.5) | R (≥4) | S (2) | S (2) | S (NEG) |
| 1047 | R (≥0.5) | R (≥8) | R (≥8) | S (0.5) | R (≥8) | S (≤0.5) | R (160) | R (≥32) | R (≥4) | S (1) | S (1) | R (POS) |
| 1069 | R (≥0.5) | R (≥8) | R (≥8) | S (0.5) | R (≥8) | R (≥16) | R (80) | R (≥32) | R (≥4) | S (2) | S (1) | R (POS) |
| 1091 | R (≥0.5) | R (≥8) | R (≥8) | S (≤0.25) | R (≥8) | R (≥16) | R (160) | R (≥32) | R (≥4) | S (1) | S (1) | R (POS) |
| 1103 | R (≥0.5) | R (≥8) | R (≥8) | S (0.5) | R (≥8) | S (≤0.5) | R (80) | R (≥32) | R (≥4) | S (1) | S (2) | R (POS) |
| 1126 | R (≥0.5) | R (≥8) | R (≥8) | S (≤0.25) | S (≤0.12) | R (≥16) | R (160) | S (≤0.5) | R (≥4) | S (1) | S (2) | R (POS) |
| 1154 | R (≥0.5) | R (≥8) | R (≥8) | R (≥4) | R (≥8) | S (≤0.5) | R (160) | R (≥32) | R (≥4) | S (2) | S (1) | R (POS) |
| 1161 | R (≥0.5) | R (≥8) | S (≤0.25) | S (≤0.25) | S (≤0.12) | S (≤0.5) | R (≥320) | S (≤0.5) | R (≥4) | S (1) | S (2) | R (POS) |
| 4201 | R (≥0.5) | R (≥8) | S (≤0.25) | S (≤0.25) | S (≤0.12) | S (≤0.5) | S (≤10) | S (≤0.5) | R (≥4) | S (1) | S (1) | R (POS) |
| 4202 | R (≥0.5) | R (≥8) | R (≥8) | S (≤0.25) | S (≤0.12) | S (≤0.5) | R (160) | S (≤0.5) | R (≥4) | S (2) | S (1) | R (POS) |
| 4204 | R (0.25) | R (≥8) | R (≥8) | S (0.5) | R (4) | R (8) | R (80) | R (≥32) | R (≥4) | S (2) | S (2) | R (POS) |
| 4205 | R (≥0.5) | R (≥8) | R (≥8) | S (0.5) | R (4) | R (≥16) | R (160) | S (≤0.5) | R (≥4) | S (1) | S (1) | R (POS) |
| 4206 | R (≥0.5) | R (≥8) | R (≥8) | S (≤0.25) | R (≥8) | R (≥16) | R (80) | R (1) | R (≥4) | S (1) | S (1) | R (POS) |
| 4208 | R (≥0.5) | R (≥8) | R (≥8) | S (0.5) | R (≥8) | R (≥16) | R (160) | S (≤0.5) | R (≥4) | S (1) | S (1) | R (POS) |
| 4210 | R (≥0.5) | S (≤0.25) | S (≤0.25) | S (≤0.25) | S (≤0.12) | S (≤0.5) | R (160) | S (≤0.5) | R (≥4) | S (1) | S (1) | R (POS) |
| 4211 | R (≥0.5) | R (≥8) | R (≥8) | S (≤0.25) | S (≤0.12) | R (≥16) | R (≥320) | S (≤0.5) | R (≥4) | S (1) | S (1) | R (POS) |
| 4212 | R (≥0.5) | R (≥8) | R (≥8) | S (≤0.25) | R (≥8) | S (≤0.5) | R (160) | S (≤0.5) | R (≥4) | S (1) | S (1) | R (POS) |
| 4214 | R (≥0.5) | R (≥8) | S (≤0.25) | S (≤0.25) | S (≤0.12) | R (≥16) | S (20) | S (≤0.5) | R (≥4) | S (1) | S (2) | R (POS) |

BP: benzylpenicillin, EM: erythromycin, CM: clindamycin, Q/D: quinupristin/dalfopristin, LE: levofloxacin, GM: gentamicin, TS: trimethoprim / sulfamethoxazole, RI: rifampicin, OX: oxacillin, LI: linezolid, VM: vancomycin, CX: cefoxitin, MIC: minimum inhibitory concentration, Interp.: interpretation, R: resistant, S: sensitive.
